# Psychosocial risks for constipation and soiling in primary school children

**DOI:** 10.1007/s00787-018-1162-8

**Published:** 2018-05-10

**Authors:** Carol Joinson, Mariusz T. Grzeda, Alexander von Gontard, Jon Heron

**Affiliations:** 1 Population Health Sciences, Bristol Medical School, University of Bristol, Oakfield House, Oakfield Grove, Clifton, Bristol, BS8 2BN UK; 2 Department of Child and Adolescent Psychiatry, Saarland University Hospital, Homburg, Germany

**Keywords:** Psychosocial problems, Constipation, Soiling, Prospective cohort, Latent class, ALSPAC

## Abstract

To examine prospective associations between psychosocial problems and childhood constipation and soiling. We used latent classes of constipation and soiling (‘constipation alone’, ‘soiling alone’, ‘constipation with soiling’) extracted from longitudinal maternally reported data on constipation (4–10 years) and soiling (4–9 years) from 8435 children (4353 males, 4082 females) from the ALSPAC cohort. We examined the association between maternally reported psychosocial problems at 2–3 years (difficult temperament, behaviour/emotional problems, temper tantrums, behavioural sleep problems and stressful events) and the latent classes using multinomial logistic regression adjusted for a range of confounders relating to the child and family (reference category = normative latent class with very low probability of constipation/soiling). Difficult temperament and emotional/behaviour problems were associated with increased odds of constipation and soiling. Associations were generally strongest for ‘constipation with soiling’, e.g. difficult mood: 1.42 (1.23–1.64); behaviour problems: 1.48 (1.28–1.71); temper tantrums: 1.89 (1.34–2.65); lack of a regular sleep routine 2.09 (1.35–3.25). Stressful life events were associated with constipation alone [1.23 (1.12–1.36)] and constipation with soiling [1.32 (1.14–1.52)], but not soiling alone. Additional comparisons of the non-normative latent classes provided evidence for differential associations with the risk factors, e.g. frequent temper tantrums were associated with a greater than twofold increase in the odds of constipation with soiling versus constipation alone. Psychosocial problems in early childhood are risk factors for constipation and soiling at school age. An increased understanding of early risk factors for constipation and soiling could aid the identification of children who require treatment.

**Electronic supplementary material:**

The online version of this article (10.1007/s00787-018-1162-8) contains supplementary material, which is available to authorized users.

## Introduction

Constipation and soiling are common in childhood [1, 2] and the majority of cases are functional. The principal precipitant of acute constipation in most children is painful hard stools [3]. Constipation becomes chronic through a vicious cycle of stool retention, painful defecation, stool avoidance and hardening of retained stool through fluid reabsorption. Soiling is a common consequence of chronic constipation [3, 4], but an estimated 20% of children experience soiling with no underlying constipation [5]. Risk factors for onset and maintenance of functional constipation and soiling are believed to involve a complex interaction of genetic, biological and neurological factors, as well as diet and toilet training. Psychosocial factors are also thought to play a role, since clinicians often observe that children with constipation and/or soiling suffer from psychological problems and stress. Cross-sectional studies report high rates of emotional/behaviour problems [2, 6–12] and stressful events [13] in children with constipation and soiling. A few studies have compared rates of psychosocial problems in children with constipation alone, soiling alone (non-retentive) and constipation with soiling (retentive). Higher levels of behaviour problems were found in children with constipation plus soiling than constipation alone [14] and children with non-retentive soiling were reported to have higher rates of psychological symptoms than those with retentive soiling [15]. A study comparing rates of behaviour problems in children with constipation and those with soiling without constipation, found no difference between the two groups [5]. Very little is known about the aetiology of non-retentive soiling, but psychological disturbance is thought to be a contributing factor [16]. No prospective studies have examined whether psychosocial problems are more strongly associated with non-retentive versus retentive soiling. The current study uses data on constipation and soiling from a birth cohort to examine prospective associations between psychosocial problems in early childhood and different patterns of constipation and soiling at school age.

## Methods

### Participants

The sample comprised participants from the Avon Longitudinal Study of Parents and Children (ALSPAC). Detailed information is available at (http://www.bristol.ac.uk/alspac), including a fully searchable data-dictionary (http://www.bris.ac.uk/alspac/researchers/data-access/data-dictionary). Pregnant women resident in the former Avon Health Authority in SW England, having an estimated date of delivery between 1/4/91 and 31/12/92 were invited to take part, resulting in a cohort of 14,541 pregnancies [17]. Of the 13,978 singletons/twins alive at 1 year, a small number of participants withdrew consent (*n* = 24) leaving a starting sample of 13,954. Ethical approval was obtained from the ALSPAC Law and Ethics committee and local research ethics committees.

### Outcomes: latent classes of constipation and soiling in childhood

When children were 4½, 5½, 6½, 7½ and 9½ (hereafter referred to as 4–9 years) parents were asked: “How often usually does your child dirty his/her pants during the day?” Options were: ‘Never’; ‘Occasional accidents but less than once a week’; ‘About once a week’; ‘2–5 times a week’; ‘Nearly every day’; and ‘More than once a day’. Parents were also asked about their child’s constipation at 4 years 9 months, 5 years 9 months, 6 years 9 months, 7 years 7 months, 8 years 7 months, 10 years 8 months (hereafter referred to as 4–10 years): “Has he/she had any constipation in the past 12 months?” Options were: ‘Yes, and saw a doctor’; ‘Yes, but did not see the doctor’, and ‘No, did not have’. These data were used to extract four latent classes of constipation and soiling during childhood using longitudinal latent class analysis (LLCA) [18]. The ‘normative’ class (very low probability of constipation or soiling during childhood) had the highest prevalence (74.5%) followed by ‘constipation alone’ (13.2%), ‘soiling alone’ (7.5%) and ‘constipation with soiling’ (4.8%) [18].

### Exposures: psychosocial problems in early childhood

Mothers completed the Toddler Temperament Scale (TTS) [19] when study children were 2 years (we restricted our analysis to five traits that were associated with soiling in an earlier study) [20]. The Emotionality Activity Sociability (EAS) Questionnaire [21] was administered when study children were 3 years. Behaviour and emotional problems were assessed at 3½ years using the Revised Rutter Parent Scale for Preschool Children [22]. Mothers also reported the frequency of their child’s temper tantrums and behavioural sleep problems at 3½ years. Stressful events were measured using a maternally reported questionnaire (completed when the study child was 3 years 11 months) [23].

### Confounders

We adjusted for a range of confounders including sex and socio-demographic measures derived from responses to a maternal questionnaire completed during the antenatal period, containing items on social class, early parenthood, housing adequacy, maternal education, major financial difficulties, family size and the presence of a social network (see Table 1). We also adjusted for the child’s developmental level at 18 months using a questionnaire developed by ALSPAC including items from the Denver Developmental Screening Test [24] and maternal depression using the Edinburgh Postnatal Depression Scale (EPDS) [25] (when the child was 21 or 33 months depending on the timing of the exposure being examined). The model for sleep problems at 3½ years was additionally adjusted for difficult temperament traits (TTS) at 2 years.

**Table 1:** Distribution of socio-demographic, family factors and urinary incontinence in each latent class (*n* ≤ 8435 depending on risk factor)

Variable (number of missing values)^a^	Normative (%)	Constipation alone (%)	Soiling alone (%)	Constipation with soiling (%)	Total (%)	*P* value
Sex (0)						
Male	51.7	42.0	64.2	58.2	51.6	< 0.001
Social class^b^ (678)						
Manual	15.2	14.5	16.7	14.9	15.2	0.739
Early parenthood (0)						
< 19 years	4.7	4.5	2.9	4.8	4.6	0.338
Maternal educational qualifications (211)						
None	23.6	21.1	23.8	21.2	23.2	0.321
Housing adequacy^c^ (109)						
No	4.8	4.3	4.2	4.9	4.7	0.870
Social network^d^ (226)						
No	8.8	9.9	11.6	12.4	9.3	0.036
Major financial difficulties (418)						
Yes	7.5	9.3	12.1	11.3	8.1	< 0.001
Family size (108)						
≥ 3 children	1.0	0.5	0.9	1.5	1.0	0.293
Maternal depression^e^ (child aged 21 months) (585)						
Yes	8.2	10.8	12.9	15.6	9.0	< 0.001
Maternal depression (child aged 33 months) (698)						
Yes	10.5	14.2	16.6	19.3	11.5	< 0.001
Urinary incontinence at 7½ years						
Bedwetting (770)	13.8	14.8	31.1	25.0	15.3	< 0.001
Daytime wetting (765)	5.6	6.8	30.4	28.2	7.9	< 0.001
Combined (day and night) wetting (777)	17.2	19.1	46.4	40.1	19.8	< 0.001

We obtained these distributions by assigning each participant to their most likely class (modal assignment), generating individual probabilities of class membership and deriving conditional distributions of the variables within classes

^a^The proportion of the sample with missing data for socio-demographic variables was small, accounting for less than 5% of the overall sample, except social class (8% missing). Proportions of missing data were higher for maternal depression and urinary incontinence, but did not exceed 10%

^b^Manual social class comprises partly or unskilled occupations

^c^Housing adequacy: ‘no’ comprises crowding, periods of homelessness, poor living conditions, major defects/infestation in home

^d^Social network: ‘no’ comprises lack of emotional/practical/financial support

^e^Maternal depression: the EPDS was dichotomized at the standard cut-off (score > 12) to indicate probable depression

### Statistical modelling

We estimated the association between the risk factors and class membership using a series of multinomial logistic regression models employing the normative latent class as the baseline category for the outcome, before re-parameterizing to derive comparisons across the other outcome classes. Parameter estimates were obtained using the “Modal ML” 3-step method [26] implemented in Mplus. This has been shown to produce less-biased estimates than traditional three-step methods such as probability weighting, whilst avoiding the problem of covariates impacting on the measurement model itself [27]. Bias-adjusted estimates were obtained using the Mplus “auxiliary (r3step)” command.

## Results

There were 10,450 children (5384 boys and 5066 girls) with at least one non-missing time point for both constipation and soiling data. For this analysis, we focussed on the sample of 8435 (4353 boys and 4082 girls) with constipation and soiling data from at least three non-missing time points (4931 had complete data on constipation and soiling at all time points).

Table 1 shows the distribution of socio-demographic, family factors and comorbid urinary incontinence in each latent class. Females had a higher rate of constipation alone, whilst rates of constipation with soiling and soiling alone were higher in males. Rates of major financial difficulties, maternal depression (and to a lesser extent, lack of a social network) were higher in the atypical classes compared with the normative class. Children with soiling alone had the highest rate of urinary incontinence. Rates of urinary incontinence in children with constipation alone were similar to the normative class.

Table 2 presents the adjusted odds ratios for the associations between the psychosocial risk factors and membership of the latent classes of constipation and soiling (the unadjusted odds ratios are in table S1, available online).

**Table 2:** Adjusted odds ratios and 95% confidence intervals for the association between psychosocial problems and classes of constipation and soiling

	Constipation alone	Soiling alone	Constipation with soiling	*P* value
	OR (95% CI)	OR (95% CI)	OR (95% CI)	
Temperament at 2 years (TTS)^a^				
Activity (*n* = 6649)	1.08 (0.98–1.19)	1.17 (1.02–1.34)	1.09 (0.94–1.27)	0.032
Adaptability (*n* = 6631)	1.14 (1.03–1.25)	1.16 (1.01–1.32)	1.21 (1.06–1.38)	0.001
Intensity (*n* = 6647)	1.17 (1.06–1.28)	1.07 (0.94–1.23)	1.16 (1.01–1.32)	0.001
Mood (*n* = 6652)	1.28 (1.17–1.41)	1.27 (1.10–1.46)	1.42 (1.23–1.64)	< 0.001
Persistence (*n* = 6648)	1.09 (0.99–1.20)	1.02 (0.90–1.16)	1.19 (1.03–1.37)	0.040
Temperament at 3 years (EAS)^a^				
Emotionality (*n* = 6581)	1.16 (1.06–1.28)	1.16 (1.01–1.32)	1.29 (1.12–1.49)	< 0.001
Activity (*n* = 6582)	0.90 (0.82–0.99)	1.00 (0.87–1.15)	0.81 (0.71–0.93)	0.002
Shyness (*n* = 6582)	1.11 (1.01–1.22)	0.89 (0.77–1.02)	1.03 (0.89–1.21)	0.026
Sociability (*n* = 6579)	1.00 (0.91–1.10)	1.05 (0.92–1.20)	1.01 (0.87–1.17)	0.910
Behaviour and emotional problems at 3½ years (Revised Rutter scale)^a^ (*n* = 6561)				
Emotional	1.25 (1.14–1.38)	1.15 (1.01–1.30)	1.32 (1.16–1.51)	< 0.001
Behaviour	1.26 (1.14–1.39)	1.43 (1.25–1.63)	1.48 (1.28–1.71)	< 0.001
Conduct	1.13 (1.02–1.25)	1.32 (1.16–1.51)	1.23 (1.06–1.42)	< 0.001
Hyperactivity	1.02 (0.93–1.13)	1.18 (1.04–1.35)	1.09 (0.93–1.27)	0.046
Prosocial	1.06 (0.96–1.18)	1.20 (1.05–1.37)	1.11 (0.95–1.30)	0.020
Temper tantrums at 3½ years				
Once a day or most days (*n* = 6510)	0.89 (0.67–1.18)	1.26 (0.90–1.78)	1.89 (1.34–2.65)	< 0.001
Sleep problems at 3½ years				
No regular sleep routine (*n* = 6359)	1.54 (1.09–2.18)	0.76 (0.40–1.44)	2.09 (1.35–3.25)	< 0.001
Refused to go to bed (*n* = 6372)	1.47 (1.21–1.80)	1.35 (1.03–1.78)	1.33 (0.96–1.83)	< 0.001
Difficulty going to sleep (*n* = 6372)	1.49 (1.22–1.81)	1.39 (1.06–1.83)	1.50 (1.10–2.05)	< 0.001
Nightmares (*n* = 6372)	1.53 (1.25–1.86)	1.16 (0.87–1.55)	1.51 (1.10–2.06)	< 0.001
Gets up after put to bed (*n* = 6372)	1.21 (0.99–1.48)	1.54 (1.17–2.03)	1.51 (1.10–2.08)	< 0.001
Woken in the night (*n* = 6372)	1.37 (1.10–1.71)	1.53 (1.10–2.13)	1.37 (0.96–1.97)	< 0.001
Exposure to stressful life events between 2½ years and 3 years 11 months^a^				
Stressful life events score (*n* = 6500)	1.23 (1.12–1.36)	1.10 (0.95–1.27)	1.32 (1.14–1.52)	< 0.001

The analysis of temperament (TTS) and stressful events was adjusted for maternal depression at 21 months whilst all the other analyses were adjusted for maternal depression at 33 months. The analyses of sleep-related behaviours were additionally adjusted for the temperament traits (TTS). Revised Rutter Scale: High levels of psychological problems are indicated by high scores on emotional problems, conduct problems and hyperactivity and low scores on the prosocial behaviour scale. Odds ratios were derived in relation to the normative latent class (used as the reference category). The sample size for each analysis is shown in brackets after the variable name. Number of missing values on the psychosocial risk factors: TTS (*n* = 441–472); EAS (*n* = 407–414); Revised Rutter (*n* = 396); temper tantrums (*n* = 452); sleep problems (*n* = 414–433); stressful events (*n* = 491). The proportion of the sample with missing data for psychosocial variables was around 5% of the overall sample

^a^Continuous variables: increase in odds of membership to each latent class per 1 SD increase in the score

### Early temperament: TTS at 2 years

The strongest associations were found for mood; odds ratios were highest for constipation with soiling and adjusting for confounders made little difference. Adaptability was also associated with all three constipation/soiling classes and associations remained in the adjusted models. There was evidence for associations with intensity (constipation alone and constipation with soiling) and persistence (constipation with soiling). Activity was associated with soiling alone, but not the other classes. The most important confounders were maternal depression, developmental delay and major financial difficulties. Child’s sex was an additional confounder in the analysis of the association between the temperament traits of adaptability and mood and soiling alone.

### Early temperament: EAS at 3 years

Emotionality was associated with all three constipation/soiling classes and associations remained in the adjusted models. There was some evidence for associations with activity (decreased odds of constipation alone and constipation with soiling classes) and shyness (increased odds of constipation alone).

### Behaviour and emotional problems: Revised Rutter at 3½ years

Behaviour, conduct and emotional problems were associated with all three constipation/soiling classes and associations remained in the adjusted models. It is notable that the soiling alone class was the only one that was associated with the hyperactivity and prosocial scales. Maternal depression and major financial difficulties were the most important confounders (and child’s sex was a confounder in the analysis with soiling alone).

### Temper tantrums at 3½ years

Children with frequent temper tantrums at 3½ years had increased odds of constipation with soiling, but there was no evidence of an association with the other classes.

### Behavioural sleep problems at 3½ years

There was over a twofold increase in constipation with soiling among children with no regular sleep routine at age 3½. ‘Refusal to go to bed’ and ‘difficulty going to sleep’ were associated with all three constipation/soiling classes. The most important confounders were maternal depression, major financial difficulties and mood (and child’s sex in the analysis with soiling alone).

### Stressful life events

Exposure to stressful events between 2½ years and 3 years 11 months was associated with increased odds of constipation alone and constipation with soiling classes, but not soiling alone.

Additional comparisons of the non-normative latent classes: We additionally examined whether the risk factors distinguish between the non-normative classes by re-parameterizing the regression models (see table S2, available online). Notable findings were that children with higher EAS activity scores had increased odds of soiling alone versus constipation with soiling; higher EAS shyness scores were associated with increased odds of constipation alone versus soiling alone; frequent temper tantrums were associated with a greater than twofold increase in the odds of constipation with soiling versus constipation alone and finally, lack of a regular sleep routine was associated with over a twofold increase in the odds of constipation (with or without soiling) versus soiling alone.

## Discussion

Children who experienced constipation with soiling generally had the highest levels of psychosocial problems compared with the other classes; however, confidence intervals usually overlapped. Earlier studies reported higher levels of behaviour problems in children with constipation plus soiling than constipation alone [14] and higher rates of psychological symptoms in children with non-retentive soiling than retentive soiling [15], but this is not a consistent finding [5]. We found evidence that sleep problems at 3½ years were associated with constipation and soiling at school age. An earlier study found an association between sleep problems and gastrointestinal symptoms, including chronic constipation in typically developing children and in children with ASD [28]. In agreement with earlier studies [13] stressful events were associated with constipation with and without soiling, but stressful events were not associated with soiling alone. We found little evidence for associations between the constipation/soiling classes and a range of socioeconomic indicators. The exception was the rate of major financial difficulties, which was highest in the two soiling classes (with or without constipation). A US study found that soiling was more common in children from families with lower income, whilst constipation was associated with higher income [29]. It is notable that rates of urinary incontinence were highest in children with soiling alone, whilst children with constipation alone had similar rates of urinary incontinence to the normative class. Comorbidity between urinary and faecal incontinence has been reported previously [29, 30], whilst another study found higher rates of urinary incontinence in children with constipation compared to those without [31].

### Strengths and limitations

A major strength of this study is the use of latent classes extracted using repeated measures of constipation and soiling in a large cohort. A limitation is that constipation and soiling were based on parental report rather than standardised clinical criteria. Parents were asked to report whether or not their child had suffered from constipation in the past 12 months and if they had seen doctor. Seeing a doctor for constipation is not necessarily an indicator of the severity, but is more likely to be determined by other factors such as parental health literacy. Underreporting of constipation is possible since some parents may have been unaware that their child is constipated. The prevalence of constipation (18%) observed in this cohort [18], however, is higher than the median prevalence (8.9%) reported in a systematic review of 0- to 18-year-olds [18]. Parents were not asked about the duration of constipation or soiling at each time point, but the repeated measures of constipation and soiling in the ALSPAC cohort suggest constipation and soiling are persistent problems. It is possible that parents of children who soil in the present study were unaware that their child may be constipated, or parents might mistake the leakage of liquid stool due to faecal retention as diarrhoea. Some parents noted in the questionnaires that their child had soiled his/her pants only because of diarrhoea or inadequate wiping and they did not place their children in the soiling category. We did not include information on frequency of soiling in the latent class models and there were no data available on frequency of constipation. Our aim was to examine risk factors for constipation and soiling in the community and not to focus solely on children who meet currently established diagnostic criteria. We were unable to assess potentially important parent–offspring associations due to an absence of data on family history. Genetic factors play an important role in childhood constipation (with or without soiling) [5, 32], but parent-offspring associations for soiling alone are less strong [5]. There was no information on underlying organic causes of constipation and soiling in our sample, but the vast majority of cases are functional.

### Potential mechanisms explaining the findings

There is a strong genetic disposition for constipation [32] and most children develop functional constipation in infancy [33]. It is possible that early psychological distress is a manifestation of the pain and discomfort associated with constipation. Functional constipation is believed to be triggered by adverse experiences relating to defecation, especially painful bowel movements, which results in avoidance of defecation due to the anticipation of further pain [4]. Children with difficult temperament and emotional problems might be more prone to experiencing defecation anxiety, stool withholding and difficulties with toilet training. Earlier studies based on small clinic samples found evidence for more difficult temperament traits in children who are difficult to toilet train [34] and in children with stool toileting refusal [35]. It has, however, been argued that stool withholding is unlikely to be caused primarily by behaviour problems, since constipation often starts in infancy [33] and earlier findings show that hard and painful bowel movements usually occur before the onset of stool toileting refusal [4]. Another explanation for the findings could be a common underlying cause of emotional/behaviour problems and bowel problems. For instance, bowel problems are more common in children with autism spectrum disorder (ASD) [36] and attention deficit hyperactivity disorder (ADHD) [11] than in typically developing children and these disorders are commonly associated with high rates of emotional/behaviour problems. In our sample, ASD rates were highest in soiling alone (2.4%) and constipation with soiling (2.1%), compared with constipation alone (0.6%) and normative classes (0.5%). Rates of ADHD were also highest in soiling alone (5.7%) and constipation with soiling (5.6%), compared with constipation alone (1.6%) and normative classes (1.7%). We found that higher activity levels (TTS) and hyperactivity and lower levels of prosocial behaviour were associated with soiling alone but not the other classes. We also found that higher activity levels were associated with increased odds of soiling alone compared to constipation with soiling. It has been suggested that children who are active and/or inattentive might be less able to recognize and respond to physiological cues to defecate [11]. In addition, it has been reported that the frequency of daytime and nighttime wetting is higher in children with non-retentive soiling compared with constipation [37]. This is consistent with our findings when we examined rates of urinary incontinence at age 7½, suggesting that the former group may neglect, or be less able to recognize, cues from a full bladder or bowel. Very little is known about the aetiology of non-retentive soiling, but it has been suggested that this may be a distinct subtype with different pathophysiological mechanisms [16]. Also, in comparison to constipation and retentive soiling, genetic factors play a less important role in non-retentive soiling [5].

We also found evidence that sleep problems in early childhood are associated with constipation (with or without soiling) and soiling alone. Additional comparisons between the atypical classes revealed that the lack of a regular sleep routine was associated with over a twofold increase in the odds of constipation (with or without soiling) versus soiling alone. The pain and discomfort caused by constipation might explain this finding. An alternative explanation is that sleep problems are more common in children with emotional/behaviour problems.

This is the first prospective study to find evidence that early stressful events are associated with constipation (with or without soiling) at school age. Exposure to stressful events around the time of toilet training could potentially disrupt the toilet training process, which might lead to stool withholding and constipation, e.g. families under stress may be less responsive to their child’s toileting needs, less consistent in their toilet training strategies or employ more harsh measures to dealing with their child’s incontinence. To investigate this further, we carried out an additional analysis to examine whether early stressful events are differentially associated with early childhood constipation (at 4 years) versus later onset of constipation (after age 6). The results showed a strong association between stressful events and later onset of constipation [odds ratio 1.58 (1.40–1.78)], but no association with early onset constipation [1.12 (0.97–1.30)]. These findings suggest that stressful events do not contribute to the development of functional constipation in early childhood, but do appear to have a role in later onset constipation. It is unclear why stressful events are not associated with soiling alone. Soiling alone may be a comorbid problem that is associated with other underlying disorders (e.g. attention/activity problems) rather than exposure to stress.

## Conclusion

An increased understanding of early risk factors for constipation and soiling could aid the identification of children who require treatment, and this might reduce the adverse impacts on health-related quality of life. Parents often delay seeking treatment despite the adverse impacts of soiling and many are unaware of the potential causes, with some believing it is due to laziness or stubbornness [5]. Only a small proportion of children see a doctor for soiling [2], perhaps because parents are unaware that medical advice and treatment is available. Further research is needed to examine factors in the child’s wider environment that could maintain constipation and soiling at school age such as lack of time available for toileting, unhygienic toilets at school or fear of bullying or teasing when using the toilet [38]. The strong association between constipation/soiling and psychological problems has implications for clinicians treating these children since these co-morbidities can adversely affect treatment outcomes [39]. Early assessment and treatment of psychological problems might improve treatment response in children with constipation and soiling.

## Electronic supplementary material

Below is the link to the electronic supplementary material.

Supplementary material 1 (DOCX 130 KB)
