# Supplementary material for: Psychosocial risks for constipation and soiling in primary school children
Source: Eur Child Adolesc Psychiatry. 2018 May 10;28(2):203–10. doi: 10.1007/s00787-018-1162-8 (PMC7019639; doi:10.1007/s00787-018-1162-8)
Supplement: Supplementary file 1 — Supplementary material 1 (DOCX 130 KB) [file 787_2018_1162_MOESM1_ESM.docx]

Table S1. Unadjusted odds ratios and 95% confidence intervals for the association between psychosocial problems and classes of constipation and soiling.

|  | **Constipation alone** | **Soiling alone** | **Constipation with soiling** |  |
| --- | --- | --- | --- | --- |
|  | OR [95% CI] | OR [95% CI] | OR [95% CI] | P-value |
| *Temperament at 2 years (TTS)* | |  |  |  |
| Activity (n= 7,994) | 1.06 [0.98-1.16] | 1.17 [1.04-1.32] | 1.09 [0.95-1.25] | 0.020 |
| Adaptability (n= 7,963) | 1.12 [1.03-1.22] | 1.24 [1.10-1.39] | 1.22 [1.09-1.38] | <0.001 |
| Intensity (n= 7,989) | 1.21 [1.11-1.32] | 1.07 [0.95-1.21] | 1.20 [1.06-1.35] | <0.001 |
| Mood (n= 7,994) | 1.27 [1.16-1.38] | 1.31 [1.16-1.48] | 1.43 [1.27-1.61] | <0.001 |
| Persistence (n= 7,989) | 1.08 [0.99-1.18] | 1.15 [1.03-1.28] | 1.27 [1.12-1.44] | <0.001 |
|  |  |  |  |  |
| *Temperament at 3 years (EAS)* | |  |  |  |
| Emotionality (n= 8,025) | 1.24 [1.14-1.35] | 1.17 [1.05-1.32] | 1.31 [1.17-1.48] | <0.001 |
| Activity (n= 8,028) | 0.90 [0.83-0.98] | 0.97 [0.85-1.11] | 0.81 [0.72-0.91] | <0.001 |
| Shyness (n= 8,025) | 1.13 [1.03-1.23] | 0.92 [0.82-1.04] | 1.05 [0.92-1.21] | 0.011 |
| Sociability (n= 8,021) | 0.98 [0.90-1.07] | 1.17 [1.04-1.32] | 1.04 [0.91-1.19] | 0.052 |
|  |  |  |  |  |
| *Behaviour & emotional problems at 3½ years (Revised Rutter scale)* (n= 8,039) | | | |  |
| Emotional | 1.27 [1.17-1.38] | 1.20 [1.07-1.35] | 1.41 [1.24-1.59] | <0.001 |
| Behaviour | 1.25 [1.15-1.36] | 1.55 [1.39-1.74] | 1.64 [1.46-1.85] | <0.001 |
| Conduct | 1.10 [1.01-1.21] | 1.38 [1.24-1.54] | 1.33 [1.18-1.50] | <0.001 |
| Hyperactivity | 1.06 [0.97-1.15] | 1.29 [1.15-1.44] | 1.23 [1.07-1.42] | <0.001 |
| Prosocial | 1.00 [0.91-1.09] | 1.37 [1.22-1.54] | 1.22 [1.07-1.40] | <0.001 |
|  |  |  |  |  |
| *Temper tantrums at 3½ years* (n= 7,983) | |  |  |  |
| Once a day or most days | 1.05 [0.82-1.33] | 1.41 [1.04-1.92] | 2.00 [1.48-2.68] | <0.001 |
|  |  |  |  |  |
| *Sleep problems at 3½ years* | |  |  |  |
| No regular sleep routine (n= 8,002) | 1.46 [1.08-1.98] | 0.76 [0.43-1.34] | 2.21 [1.52-3.23] | <0.001 |
| Refused to go to bed (n= 8,021) | 1.51 [1.27-1.79] | 1.32 [1.04-1.67] | 1.53 [1.18-1.98] | <0.001 |
| Difficulty going to sleep (n= 8,021) | 1.53 [1.29-1.82] | 1.40 [1.10-1.78] | 1.58 [1.22-2.06] | <0.001 |
| Nightmares (n= 8,021) | 1.64 [1.38-1.95] | 1.17 [0.91-1.50] | 1.53 [1.17-1.99] | <0.001 |
| Gets up after put to bed (n= 8,021) | 1.33 [1.12-1.58] | 1.62 [1.28-2.06] | 1.46 [1.07-1.99] | <0.001 |
| Woken in the night (n= 8,021) | 1.39 [1.14-1.70] | 1.62 [1.21-2.17] | 1.46 [1.07-1.99] | <0.001 |
|  |  |  |  |  |
| *Exposure to stressful life events between 2½ years & 3 years 11 months* (n= 7,944) | | | |  |
| Stressful life events score | 1.28 [1.17-1.38] | 1.08 [0.96- 1.22] | 1.36 [1.21-1.54] | <0.001 |

The sample size for each analysis is shown in brackets after the variable name.

Table S2. Post-hoc comparisons examining whether the psychosocial risk factors discriminate between the atypical classes

|  | **Unadjusted models** | | | | **Adjusted models** | | | |
| --- | --- | --- | --- | --- | --- | --- | --- | --- |
|  | **Soiling alone vs. constipation & soiling** | | **Constipation & soiling vs.**  **constipation alone** | **Constipation alone vs.**  **soiling alone** | **Soiling alone vs. constipation & soiling** | **Constipation & soiling vs. constipation alone** | | **Constipation alone vs.**  **soiling alone** |
|  | OR [95% CI] | | OR [95% CI] | OR [95% CI] | OR [95% CI] | OR [95% CI] | | OR [95% CI] |
| *Temperament at 2 years (TTS)^1^* | | |  |  |  |  | | |
| Activity | 1.08 [0.89-1.30] | | 1.02 [0.86-1.21] | 0.91 [0.79-1.04] | 1.07 [0.87-1.32] | 1.01 [0.84-1.21] | | 0.92 [0.80-1.07] |
| Adaptability | 1.01 [0.86-1.20] | | 1.09 [0.94-1.26] | 0.91 [0.79-1.03] | 0.96 [0.79-1.16] | 1.06 [0.90-1.25] | | 0.98 [0.85-1.14] |
| Intensity | 0.90 [0.75-1.07] | | 0.99 [0.85-1.15] | 1.13 [0.98-1.29] | 0.93 [0.76-1.12] | 0.99 [0.84-1.17] | | 1.09 [0.94-1.26] |
| Mood | 0.92 [0.77-1.09] | | 1.13 [0.97-1.31] | 1.03 [0.90-1.18] | 0.89 [0.73-1.10] | 1.11 [0.93-1.31] | | 1.01 [0.87-1.18] |
| Persistence | 0.90 [0.76-1.07] | | 1.18 [1.01-1.37] | 0.94 [0.83-1.07] | 0.86 [0.71-1.05] | 1.09 [0.91-1.30] | | 1.07 [0.92-1.24] |
|  |  | |  |  |  |  | |  |
| *Temperament at 3 years (EAS)^1^* | | |  |  |  |  | | |
| Emotionality | 0.89 [0.75-1.06] | | 1.06 [0.91-1.22] | 1.06 [0.93-1.21] | 0.90 [0.73-1.09] | 1.11 [0.93-1.32] | | 1.01 [0.87-1.17] |
| Activity | 1.20 [1.00-1.44] | | 0.90 [0.78-1.04] | 0.92 [0.80-1.07] | 1.23 [1.01-1.51] | 0.90 [0.76-1.06] | | 0.90 [0.77-1.05] |
| Shyness | 0.88 [0.72-1.07] | | 0.93 [0.79-1.10] | 1.22 [1.06-1.40] | 0.86 [0.69-1.07] | 0.93 [0.77-1.12] | | 1.25 [1.07-1.46] |
| Sociability | 1.13 [0.93-1.37] | | 1.06 [0.90-1.25] | 0.84 [0.73-0.96] | 1.04 [0.84-1.28] | 1.01 [0.84-1.21] | | 0.95 [0.82-1.11] |
|  |  | |  |  |  |  | |  |
| *Behaviour & emotional problems at 3½ years (Revised Rutter scale) ^1^* | | | |  |  |  | |  |
| Emotional | 0.86 [0.72-1.01] | | 1.11 [0.95-1.29] | 1.06 [0.93-1.20] | 0.87 [0.72-1.04] | 1.05 [0.89-1.24] | | 1.09 [0.95-1.26] |
| Behaviour | 1.04 [0.88-1.22] | | 1.31 [1.13-1.52] | 0.80 [0.71-0.91] | 1.08 [0.88-1.32] | 1.09 [0.91-1.31] | | 0.85 [0.74-0.99] |
| Conduct | 1.04 [0.86-1.27] | | 1.21 [1.04-1.40] | 0.80 [0.70-0.91] | 1.09 [0.88-1.35] | 1.06 [0.87-1.28] | | 0.87 [0.75-1.00] |
| Hyperactivity | 1.12 [0.93-1.35] | | 1.16 [0.98-1.38] | 0.82 [0.72-0.94] | 1.08 [0.87-1.33] | 1.05 [0.87-1.27] | | 0.89 [0.76-1.03] |
| Prosocial | 0.95 [0.80-1.11] | | 1.23 [1.04-1.45] | 0.73 [0.64-0.83] | 0.96 [0.79-1.17] | 1.18 [0.99-1.41] | | 0.88 [0.76-1.02] |
|  |  | |  |  |  |  | |  |
| *Temper tantrums at 3½ years^1^* | | |  |  |  |  | | |
| Once a day or most days | 0.71 [0.46-1.09] | | 1.90 [1.30-2.80] | 0.74 [0.52-1.05] | 0.67 [0.41-1.10] | 2.11 [1.35-3.30] | | 0.71 [0.47-1.06] |
|  |  | |  |  |  |  | |  |
| *Sleep problems at 3½ years^2^* | | |  |  |  |  | | |
| No regular sleep routine | 0.34 [0.17-0.70] | | 1.52 [0.94-2.46] | 1.93 [1.06-3.51] | 0.36 [0.16-0.81] | 1.36 [0.78-2.37] | 2.01 [1.04-3.90] | |
| Refused to go to bed | 0.86 [0.60-1.24] | | 1.02 [0.74-1.39] | 1.14 [0.87-1.49] | 1.02 [0.66-1.58] | 0.90 [0.61-1.32] | 1.09 [0.80-1.48] | |
| Difficulty going to sleep | 0.89 [0.61-1.28] | | 1.03 [0.75-1.42] | 1.09 [0.84-1.43] | 0.92 [0.60-1.41] | 1.01 [0.70-1.46] | 1.07 [0.79-1.46] | |
| Nightmares | 0.76 [0.52-1.11] | | 0.93 [0.68-1.29] | 1.40 [1.06-1.85] | 0.77 [0.50-1.19] | 0.99 [0.68-1.44] | 1.31 [0.96-1.80] | |
| Gets up after put to bed | 1.02 [0.71-1.47] | | 1.19 [0.87-1.64] | 0.82 [0.63-1.07] | 1.02 [0.66-1.57] | 1.24 [0.85-1.82] | 0.79 [0.58-1.07] | |
| Woken in the night | 1.11 [0.71-1.73] | | 1.05 [0.72-1.53] | 0.86 [0.62-1.19] | 1.12 [0.67-1.86] | 1.00 [0.65-1.54] | 0.89 [0.62-1.29] | |
|  |  | |  |  |  |  |  | |
| *Exposure to stressful life events between 2½ years & 3 years 11 months* | | | | |  |  | | |
| Stressful life events score | | 0.79 [0.67-0.94] | 1.07 [0.93-1.24] | 1.18 [1.03-1.34] | 0.83 [0.68-1.02] | 1.07 [0.90-1.27] | 1.12 [0.96-1.31] | |
